# Statistical analysis plan for the motor neuron disease systematic multi-arm adaptive randomised trial (MND-SMART)

**DOI:** 10.1186/s13063-022-07007-z

**Published:** 2023-01-16

**Authors:** Richard A. Parker, Christopher J. Weir, Tra My Pham, Ian R. White, Nigel Stallard, Mahesh K. B. Parmar, Robert J. Swingler, Rachel S. Dakin, Suvankar Pal, Siddharthan Chandran

**Affiliations:** 1 Edinburgh Clinical Trials Unit, Usher Institute, University of Edinburgh, Edinburgh, UK; 2 Medical Research Council Clinical Trials Unit at UCL, Institute of Clinical Trials and Methodology, University College London, London, UK; 3 Statistics and Epidemiology, Division of Health Sciences, Warwick Medical School, University of Warwick, Coventry, UK; 4 Euan Macdonald Centre for Motor Neuron Disease Research, University of Edinburgh, Edinburgh, EH16 4SB UK; 5 Centre of Clinical Brain Sciences, University of Edinburgh, Edinburgh, EH16 4SB UK; 6 Anne Rowling Regenerative Neurology Clinic, University of Edinburgh, Edinburgh, EH16 4SB UK; 7 UK Dementia Research Institute Edinburgh, University of Edinburgh, Edinburgh, EH16 4SB UK

**Keywords:** Statistical analysis plan, Motor neuron disease, MAMS trial, Multi-arm, Platform trial

## Abstract

**Background:**

MND-SMART is a platform, multi-arm, multi-stage, multi-centre, randomised controlled trial recruiting people with motor neuron disease. Initially, the treatments memantine and trazodone will each be compared against placebo, but other investigational treatments will be introduced into the trial later. The co-primary outcomes are the Amyotrophic Lateral Sclerosis Functional Rating Scale Revised (ALS-FRS-R) functional outcome, which is assessed longitudinally, and overall survival.

**Methods:**

Initially in MND-SMART, participants are randomised 1:1:1 via a minimisation algorithm to receive placebo or one of the two investigational treatments with up to 531 to be randomised in total. The comparisons between each research arm and placebo will be conducted in four stages, with the opportunity to cease further randomisations to poorly performing research arms at the end of stages 1 or 2. The final ALS-FRS-R analysis will be at the end of stage 3 and final survival analysis at the end of stage 4. The estimands for the co-primary outcomes are described in detail. The primary analysis of ALS-FRS-R at the end of stages 1 to 3 will involve fitting a normal linear mixed model to the data to calculate a mean difference in rate of ALS-FRS-R change between each research treatment and placebo. The pairwise type 1 error rate will be controlled, because each treatment comparison will generate its own distinct and separate interpretation. This publication is based on a formal statistical analysis plan document that was finalised and signed on 18 May 2022.

**Discussion:**

In developing the statistical analysis plan, we had to carefully consider several issues such as multiple testing, estimand specification, interim analyses, and statistical analysis of the repeated measurements of ALS-FRS-R. This analysis plan attempts to balance multiple factors, including minimisation of bias, maximising power and precision, and deriving clinically interpretable summaries of treatment effects.

**Trial registration:**

EudraCT Number, 2019–000099-41. Registered 2 October 2019, https://www.clinicaltrialsregister.eu/ctr-search/search?query=mnd-smart

ClinicalTrials.gov, NCT04302870. Registered 10 March 2020.

**Supplementary Information:**

The online version contains supplementary material available at 10.1186/s13063-022-07007-z.

## Background

Motor neuron disease (MND) is an incurable neurodegenerative disorder, which causes progressive paralysis affecting the limbs, speech, swallowing, and respiratory muscles. There are approximately 5000 people living with MND in the United Kingdom (UK) at any time [1]. Average survival is 2 to 3 years after diagnosis with a third of people dying within a year. The only licensed drug in the UK is riluzole, approved by the National Institute for health and Care Excellence in 2001, that prolongs survival by 2–3 months [2]. There is a pressing need for effective disease modifying treatments in MND.

The aim of this clinical trial, called Motor Neuron Disease Systematic Multi-Arm Adaptive Randomised Trial (MND-SMART), is to identify medicines which improve outcomes for people living with MND. We plan to evaluate numerous investigational treatments starting with repurposed medicines over many years using a multi-arm multi-stage trial platform design [3, 4]. Combining multi-arm, multi-stage, and repurposing is a cost and time efficient trial design, especially in rare conditions such as MND. The trial will be conducted in four stages and will generally only continue to the next stage if the results of each stage meet pre-defined criteria. In stages 1–3, the primary outcome measure is Amyotrophic Lateral Sclerosis Functional Rating Scale Revised (ALS-FRS-R) [5], which will be evaluated alongside the safety and tolerability of the drugs. ALS-FRS-R is a functional outcome scale used in MND and globally recognised by regulators including the U.S. Food and Drug Administration, European Medicines Agency, and the Medicines and Healthcare products Regulatory Agency (MHRA). In stage 4, we will analyse the effect of drugs on survival. Survival will be considered as a co-primary endpoint, and a significant survival benefit will provide pivotal evidence of efficacy only if the stage 3 analysis of ALS-FRS-R is also statistically significant.

The remainder of the paper is structured as follows. In the “Trial design” section, we summarise the main features of the MND-SMART design. In the “Approach to multiplicity adjustment” section, we discuss whether adjustments for multiple comparisons are required in a multi-arm trial such as this. In the “Overall statistics principles” section, we present our analysis populations and overall statistical principles. In the “Co-primary ALS-FRS-R analysis model development” section, we present our primary analysis model and provide information regarding the background simulation work that we performed to justify its form. In the “Estimands” section, we describe our estimands for the co-primary outcomes. In the “List of statistical analyses” section, we list our planned statistical analyses, and finally we end with a short discussion in the “Discussion” section.

### Trial design

#### Overall design

MND-SMART is a multi-arm, multi-stage, platform, parallel-group, multi-centre, randomised controlled trial recruiting people with motor neuron disease. Initially, the active treatments of memantine and trazodone will each be compared against placebo, but other active treatments will be introduced into the trial later. Participants will have five appointments in the first 4 to 8 weeks to cover screening, baseline, and drug titration followed by assessments every 2 months until trial completion. Full details regarding the study can be found in the published protocol paper [6].

#### Inclusion/exclusion criteria

The inclusion criteria for this trial are very broad and encompass almost everyone with a confirmed diagnosis of MND (of any subtype), irrespective of disease duration. Exclusion criteria include specific abnormal blood tests and electrocardiogram changes, patients diagnosed with arrhythmias, associated dementia, pregnancy, severe psychiatric disorders, and alcoholism [6]. Any participants taking a medication that interacts with the active substances or excipients in the MND-SMART study medications are also excluded. The motivation of the broad inclusion criteria was to maximise the opportunity for people with MND to participate in a randomised controlled trial and generate results generalizable for this entire population. It should be noted that this approach does not affect the integrity of the comparisons being made and that the statistical analysis plan pre-specifies relevant subgroup analyses (see the “Subgroup analysis” section). The population is women and men aged over 18 with confirmed diagnosis of MND.

In a further inclusivity measure, people with MND also have the opportunity to join MND-SMART more than once, subject to one of the following conditions:

(i) The arm they were randomised to has ceased randomising further patients, and they have completed a treatment wash-out period of 1 month.
(ii) They are in the control group and all experimental arms open at the time at which they were randomised have ceased randomising further patients.
(iii) Their data has been included in a final survival-based comparison.

#### Randomisation

Participants are randomised 1:1:1 via a minimisation algorithm to receive placebo or one of the two active treatments with up to 531 (177 per arm) randomised. Minimisation is based on three factors: riluzole use, use of non-invasive ventilation (NIV) and/or gastrostomy, and long survival (more than 8 years from diagnosis at MND-SMART screening).

#### Multi-stage design

**Fig. 1:**
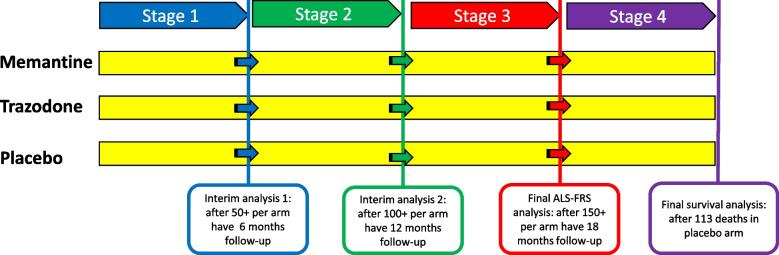
Multi-arm multi-stage study design for the MND-SMART trial

The study design is shown in Fig. 1.

Investigational treatments will be compared to placebo in up to four stages, with the opportunity to cease further randomisations to treatments that do not meet predefined continuation criteria at the end of stages 1 or 2. The first three stages will involve analysis of the co-primary outcome ALS-FRS-R and will not include data from long survivors, defined as patients having survived > 8 years from diagnosis to the baseline visit.

The rationale for excluding these patients is to reduce heterogeneity in the analysis population because it is very unlikely there will be any change in ALS-FRS-R in long survivors over this time period.

Stage 1 will be completed after 50 participants per arm (excluding long survivors) have completed a minimum of 6 months of follow-up, and stage 2 will be completed after 100 participants per arm (excluding long survivors) have completed a minimum of 12 months of follow-up. At the end of stage 1, the 95% confidence interval (CI) of the rate of change in ALS-FRS-R compared to placebo should include relative improvements of 25% or above in the observed rate of decline in the placebo arm in order for the treatment to proceed to stage 2. At the end of stage 2, the improvement in rate of change in ALS-FRS-R compared to placebo should be significant at the pairwise one-sided 10% significance level for the treatment to continue to stage 3. These guidelines for progression are non-binding criteria such that survival or other data may have a bearing on the decision to progress beyond each stage.

The stage 3 analysis of ALS-FRS will be conducted when 150 participants per arm have completed a minimum of 18 months of treatment. If the stage 3 analysis of ALS-FRS-R shows a significant benefit for an investigational treatment at the pairwise one-sided 2.5% level, then participants will continue to take their allocated treatment in stage 4 until 113 deaths have been observed in the placebo arm with a final inferential analysis of the survival co-primary outcome being performed at this point (end of stage 4).

A superiority testing framework will be used for all comparisons. No non-inferiority or equivalence testing will be performed. Further details about the interim and final analysis time points are provided in the “Defining the analysis point thresholds” section.

#### Sample size calculation

The target number of participants is based on the co-primary outcomes of ALS-FRS-R and survival. The sample size calculation was based on a simulation method, informed by ALS-FRS-R data from 3789 MND patients obtained from the Pooled Resource Open-Access ALS Clinical Trials (PRO-ACT) Open Access ALS Clinical Trial database [7, 8]. The PRO-ACT database suggested a decrease in ALS-FRS-R score of 0.95 per month in the control group.

At the first interim analysis, the probability of ceasing randomisation to an active treatment arm is 23% if there is no real difference between that arm and placebo and 2% probability if there is a true 25% reduction in the average decrease of ALS-FRS-R in that arm.

A sample size of 100 participants per arm excluding long survivors at the second interim analysis provides 86% probability of the treatment arm continuing beyond the stage 2 analysis into stage 3, assuming a true 25% reduction in the rate of decline of ALS-FRS-R.

A sample size of 150 per arm excluding long survivors provides an overall 83% probability of obtaining a significant result at the end of stage 3, after all interim analyses have been completed, assuming a true 25% reduction in the rate of decline of ALS-FRS-R and a one-sided 2.5% significance level.

For the survival co-primary outcome, 113 deaths per arm provides 90% power to detect a hazard ratio of 0.65 (one-sided 2.5% significance level). All participants, including long survivors at baseline, will be included in the primary analysis of survival. To avoid inflation of type 1 error, this analysis will only provide pivotal evidence of efficacy conditional on a statistically significant result for the final analysis of ALS-FRS at the end of stage 3. Otherwise, we will interpret the survival analysis as exploratory only.

A total of 177 patients will be randomised to each treatment arm (531 patients in total) to allow for a drop-out rate of approximately 10% and for the exclusion of 5% long survivors from the analysis of ALS-FRS-R.

### Approach to multiplicity adjustment

In early versions of the protocol (versions 1 to 5, up to 1 March 2021), we specified that we would control the overall familywise error rate (FWER) with respect to multiple treatment comparisons, given the multi-arm trial design. However, in these early versions of the protocol, we wrote that “At present there is not a clear consensus on whether family-wise or pairwise error must be controlled in multi-arm multi-stage trials. If agreement in favour of the pairwise approach emerges among clinical trialists during the course of MND-SMART, its design for future trial arms will be amended to align with this. Such a decision will be made only in response to information external to MND-SMART and will not be influenced by interim or primary analyses of MND-SMART data.” From early 2021 onwards, we felt that the balance of discussion in the literature regarding adjustment for multiple treatment comparisons in multi-arm trials had shifted, and there was an emerging consensus in favour of non-adjustment. Indeed, from 2020 onwards, there were several papers published in the literature supporting this position: Odutayo et al. [9], Parker and Weir [10], Collignon et al. [11], Choodari-Oskooei et al. [12], and, most recently, Molloy et al. [13]. Moreover, we would argue that the treatment comparisons of memantine versus placebo and trazodone versus placebo in this trial will each generate their own distinct and separate interpretations and that these will drive future research or clinical practice that is tied to specific treatment arms. We therefore decided to change the trial design from family-wise error rate control to pairwise error control (i.e. no adjustment for multiplicity). This change was made without any knowledge of unblinded data from the trial and before any interim analysis had been performed. Therefore, the updated version of the protocol (version 6.0, 1 November 2021) specifies that we will not adjust for multiplicity where this is specifically due to having multiple treatment arms of different investigational drugs, and instead we will only seek control of the pairwise type I error rates where relevant. No multiplicity adjustment will be applied to any analyses (interim or final) with respect to multiple treatment comparisons.

Nevertheless, we recognise that in all situations the decision must be made cognisant of the regulatory environment. For MND-SMART, the decision was taken following a protocol amendment with approval from the Co-sponsors NHS Lothian and University of Edinburgh, the broader ACORD collaboration (A Collaboration Of groups developing, Running and reporting multi-arm multi-stage trials in neurodegenerative Diseases), and the MHRA.

One major benefit of moving from familywise error rate control to pairwise error rate control was that it allowed us to increase statistical power considerably. The original sample size calculation was highly conservative, not only adjusting for the familywise error in the two investigational treatments currently being studied but also adjusting for up to two future treatments being introduced to the platform trial. Under familywise error rate control, our original sample size target was 200 per arm, which provided 85% power of continuing beyond the stage 2 analysis into the final stage, and an overall probability of continuing to the final stage and obtaining a significant result of 76.5% if there was a true 25% reduction in the rate of decline of ALS-FRS-R. Our simulation work showed that using the pairwise error rather than the family-wise error (involving a family of four comparisons), we could reduce the sample size needed at the end of the third stage from 200 per arm down to 150, and power would still be increased: increasing from 76.5% overall up to 83%. We also found that the number of required deaths per arm for the survival analysis reduced from 150 down to 113 to retain 90% power to detect a hazard ratio of 0.65.

### Overall statistical principles

#### Analysis populations

The analysis populations will be as follows:

(A) *Full analysis set, including long survivors*
  All randomised participants will be analysed according to their allocated treatment group, regardless of (i) the treatment or treatments actually received, (ii) the number of measurements recorded, and (iii) subsequent compliance or withdrawal. Long survivors are included in this analysis population.
(B) *Full analysis set, no long survivors*
  All randomised participants will be analysed according to their allocated treatment group, regardless of (i) the treatment or treatments actually received, (ii) the number of measurements recorded, and (iii) subsequent compliance or withdrawal. We will exclude long survivors.
(C) *Safety analysis set, including long survivors*

This analysis population will be formed of all randomised participants who received one of the study regimens, analysed according to the treatment received (memantine, trazodone or placebo). Long survivors are included in this analysis population.

The primary ALS-FRS-R analysis will be based on analysis population B (full analysis set, no long survivors), while the primary survival analysis will be based on analysis population A (full analysis set, including long survivors).

Participants randomised in error despite ineligibility at baseline will be reported in the participant flow summary but will not be included in any of the analysis populations above because no further data will be gathered on these participants. Note that this exclusion only relates to participants ineligible *at baseline*.

#### Outcome measure derivation

The co-primary outcome measure ALS Functional Rating Scale Revised (ALS-FRS-R) consists of 12 questions, each scored out of 4 points, with a maximum score of 48 and a minimum score of 0. A score of 48 represents an absence of the measured symptoms of ALS. A score of 0 represents the worst performance across each of the measured dimensions. A total score is calculated by summing the scores from each question.

The other co-primary outcome measure is patient survival (days from randomisation). Secondary outcomes are given in Table 1.

**Table 1:** Secondary outcomes and their derivation

Secondary outcome	Derivation
Cognitive function and behaviour assessed by the Edinburgh Cognitive and Behavioural ALS Screen (ECAS)	Cognitive function and behaviour will be calculated by calculating the sum total score of the 16 tasks on the ECAS. The total score will be a number from 0 to 136. The higher the total score the better the cognitive function. If any of the individual tasks are missing then so will be the total score. Domain totals will also be calculated. Carer score will be separately calculated
Anxiety and depression measured by the Hospital Anxiety and Depression Scale (including anxiety and depression sub-scales as well as overall score)	The Hospital Anxiety and Depression Scale will be calculated by summing all 14 individual items. The higher the score, the worse the anxiety and depression. Anxiety and depression subscales will also be calculated. If there are any missing individual items the subscales and total score will still be calculated provided that at least 4 items (> 50%) are recorded in each sub-domain
Respiratory function measured by forced vital capacity (FVC)	A mean value of percentage predicted FVC will be calculated
Quality of life evaluation—EQ-5D-5L index and EQ-5D visual analogue scale (EQ-5D VAS)	EQ-5D VAS requires no derivation. The EQ-5D-5L index will be calculated using the cross-walk algorithm based on EQ-5D-3L. [14, 15] A secondary analysis will use the EQ-5D-5L value set for England [16, 17]
Time from randomisation to King’s stage 4A	Days from date of randomisation to date of first rating of the patient as King’s stage 4A
Time from randomisation to King’s stage 4B	Days from date of randomisation to date of first rating of the patient as King’s stage 4B

#### Decision rules

The decision rules and significance levels for each stage analysis are shown in Table 2.

**Table 2:** List of decision rules for treatments proceeding to the next stage or concluding treatment efficacy

Stage	Analysis	Decision rule for treatment to proceed to next stage or conclude treatment efficacy
1	Interim analysis 1	The 95% confidence interval of the rate of change in ALS-FRS-R compared to placebo must include a relative improvement of 25% in the rate of decline
2	Interim analysis 2	One-sided *p*-value must be below the 10% significance level
3	Final analysis of ALS-FRS-R	One-sided *p*-value must be below the 2.5% significance level
4	Survival analysis	One-sided *p*-values for survival and stage 3 ALS-FRS-R analysis must be below the 2.5% significance level to conclude treatment efficacy

In the event that any treatment is stopped early due to lack of benefit at the end of stages 1 and 2, then a full analysis will be performed (i.e. all the analyses planned at the end of stage 3 will performed) for that specific treatment-placebo comparison, using a cleaned and locked version of the database incorporating any additional data gathered in the period between the interim analysis data cut-off and the final Trial Steering Committee decision to stop a treatment arm. An investigational treatment will be considered efficacious if significant benefit is demonstrated on both ALS-FRS-R at stage 3 and survival at stage 4.

#### Defining the analysis point thresholds

Interim analysis 1 will be conducted at the end of stage 1: when at least 50 participants in every arm (excluding long survivors) have completed a minimum of 6 months of follow-up. We will use the following criteria for determining whether a patient has completed 6 months of follow-up:

- Participant is not a long survivor at baseline
- Participant has recorded at least one post-baseline ALS-FRS-R score
- Participant was randomised at least 6 months ago
- Participant has not withdrawn from recording ALS-FRS-R, or if they have withdrawn this was at least 6 months after randomisation
- Participant did not die in the first three months after randomisation

All the above inclusion criteria need to be satisfied for the participant to be included in the numbers when determining the threshold for analysis (i.e. the point at which we achieve at least 50 per arm).

All available data will be included in each interim analysis and in particular any measurements of ALS-FRS-R taken more than 6 months after randomisation will still be included in the modelling of ALS-FRS-R rate of change over time at interim analysis 1. We will also include all patients who died even if they died within 3 months of randomisation.

Interim analysis 2 will take place at the end of stage 2: after at least 100 participants per arm (excluding long survivors) have completed a minimum of 12 months of follow-up. In this case, we will use the same inclusion criteria as above, except criterion (c) will be changed to “Participant was randomised more than 12 months ago”. Criteria (d) and (e) will remain the same.

The stage 3 analysis of ALS-FRS-R comparing an investigational treatment with placebo will be performed when 150 participants per arm have completed a minimum of 18 months of treatment. In this case, we will use the same inclusion criteria for determining the final analysis time point as above, except that criteria (c) will be changed to “Participant was randomised more than 18 months ago”. Criteria (d) and (e) will remain the same.

#### Analysis software

The planned analyses will be performed using SAS statistical software [SAS Institute, Cary, NC, USA], version 9.4 or later.

### Co-primary ALS-FRS-R analysis model development

#### Simulation work

Several different candidates for the ALS-FRS-R analysis model were compared using simulations led by TMP and IW. ALS-FRS-R and survival data were generated under three different assumptions regarding the treatment effect: (a) treatment affects neither ALS-FRS nor survival, (b) treatment affects ALS-FRS but not survival, and (c) treatment affects both ALS-FRS and survival via a shared random effect. Treatment effects on ALS-FRS were assumed to be proportional to time, while treatment effects on death rates were assumed to be constant. There is no ideal estimand for ALS-FRS-R in the presence of death [18]. We considered two estimands: the unconditional estimand (with outcomes implicitly imputed after death) and the partly conditional estimand (restricted to those still alive). Data were analysed by mixed models (targeting the unconditional estimand, which is not conditional on survival) and generalised estimating equations (GEE) models (targeting the partially conditional estimand, which is conditional on survival at each timepoint) [18–21]. For each analysis model, we also varied our assumptions regarding the treatment effect: we considered a time-specific treatment effect with no particular form assumed, treatment effect linear with time, and treatment effect proportional to time. In all cases, an unstructured covariance matrix was assumed. For performance measures, we considered bias, average model standard error, empirical standard error, coverage of 95% confidence intervals, and power, over 1000 simulation repetitions. Further details of the simulation methodology and results can be found in Supplementary File 1.

The results showed that the mixed model assuming treatment effect proportional to time had the lowest empirical standard error (was most efficient) and had the highest power. However, this was at least partly because the partly conditional estimand was slightly smaller than the unconditional estimand. We acknowledge that our data generation mechanism assumed a treatment effect proportional to time in simulation scenarios (b) and (c), which matched the statistical model.

The GEE model produced a slightly biased treatment effect on ALS-FRS-R under assumption (c) and when the GEE model specification had treatment effect proportional to time. Otherwise, the models were all unbiased for their respective estimands. Due to the increased power and precision of the mixed model assuming treatment effect proportional to time, we decided to opt for this model targeting an unconditional estimand for the ALS-FRS-R analyses. This model also had the advantage of being easier to interpret.

We therefore chose the unconditional estimand for the primary analysis which means the ALS-FRS-R outcome is compared between each treatment and placebo in a hypothetical scenario where the participants had not died. As such, there is no distinction between missing values before and after death (i.e. missing due to truncation), and the model will implicitly impute ALS-FRS-R values after death. Note also that this model is consistent with our proposed estimand (defined in the “Estimands” section), which is also unconditional and based on calculating a mean difference between slopes without reference to death. Death (survival) will be analysed as a separate co-primary endpoint.

#### Statistical model for the analysis of ALS-FRS-R data

The same statistical model will be fitted for the primary analyses at each of the interim analyses and final (end of stage 3) analysis. A normal linear mixed model incorporating an unstructured correlation matrix for the random effects will be fitted to the ALS-FRS-R outcome measurements at all timepoints including baseline, with the following explanatory variables:

(i) Measurement time (e.g. 0, 2, 4, 6 months visit) as a factor variable
(ii) Interaction between time and treatment, where treatment is a factor variable (placebo will be the reference category and with two dummy variables for the active treatments) and time is a continuous linear term
(iii) Riluzole (baseline minimisation variable)
(iv) Non-invasive ventilation (NIV) and/or gastrostomy (baseline minimisation variable)
(v) Random intercept for patient
(vi) Time as a random effect (random slope)

The model has no main effect of treatment since randomised treatment cannot affect the pre-randomisation value of the outcome. Including the main effect for time as a factor variable allows for possible non-linear time effects. Including an interaction between time and treatment, with treatment as a factor variable and time as a continuous variable, assumes treatment effect is proportional to time. The interaction term (ii) will be used to calculate the population-level summary of the estimand: the mean difference in rate of ALS-FRS-R change between each investigational treatment and placebo. Setting time as continuous in the interaction term (ii) is done to increase power and means that the testing is informed by treatment contrasts at all times, not just at individual time points.

For all stages of analysis, missing data will be dealt with as outlined in the “Dealing with Intercurrent Events and Missing data for ALS-FRS” section.

### Estimands

#### Estimand for the ALS-FRS-R co-primary outcome

Here, we define the estimand for the main analysis of the ALS-FRS-R co-primary outcome analysis in the trial, in line with the draft addendum to the International Council for Harmonisation of Technical Requirements for Pharmaceuticals for Human Use (ICH) E9 guidelines [22].

The variable of interest is ALS Functional Rating Scale (Revised) (ALS-FRS-R) measured every 2 months for a period of 18 months, and our population-level summary is the mean difference in rate of ALS-FRS-R change between each investigational treatment and placebo.

The population is women and men aged over 18 with confirmed diagnosis of MND. The main exclusion criteria have already been mentioned in the “Inclusion/exclusion criteria” section. In addition, any long survivors at baseline will be excluded from the primary analysis of ALS-FRS-R.

Table 3 shows intercurrent events that have been identified which would prevent measurement of the co-primary outcome or change the interpretation of the measured co-primary outcome. Poor adherence to allocated treatment will be handled in the same way as discontinuation of treatment (intercurrent events H to K), and we will use a “treatment policy” approach.

**Table 3:** Intercurrent events for ALS-FRS-R co-primary endpoint

Event	Strategy
A. Death before commencing randomised treatment	A **hypothetical strategy** [22] will be used assuming that deaths do not occur. In this case, only ALS-FRS-R at baseline may be available, which will be included in the analysis. If the participant did not record any ALS-FRS-R values, then the participant will be excluded from the analysis. This event is expected to be extremely rare
B. MND complication/admission. Event may include long-lasting complications such as cognitive decline leading to permanent discontinuation	The impact of this event will be minimised in the trial design through the use of telephone follow-up. In the event it does occur, the **treatment policy** strategy [22] will be used
C. Stopping riluzole during study	Riluzole affects ALS outcomes, they decline 12% slower than on no treatment. Audit data suggest that 15–20% of participants will stop riluzole during the course of MND-SMART. This event will be handled using a **treatment policy** strategy
D. Starting riluzole during study	As riluzole treatment is generally initiated close to diagnosis it is expected that few participants (< 10%) will start riluzole during MND-SMART. This event will therefore be handled by a **treatment policy** strategy
E. Deterioration in participant condition	Deterioration will occur frequently, and may vary across randomised treatment group. The trajectory of decline is expected to be observable in the ALS-FRS-R values and therefore a **treatment policy** strategy will be used
F. Concomitant illness	The **treatment policy** strategy will be used as per event B
G. Death before end of follow-up resulting in truncated data on ALS-FRS-R	A mortality rate of 25–30% is expected within 2 years of randomisation. ALS-FRS-R scores prior to death will likely reflect the imminent death. Here, a **hypothetical strategy** will be used assuming the participant has not died. Separate consideration of deaths not due to MND is not required as these will be rare
H. Discontinuation of memantine due to intolerance or adverse event	These discontinuations will mostly occur early, in about 10% of participants, who will then continue in study via telephone follow-up. The treatment policy perspective of MND-SMART means that the **treatment policy** strategy can be used to handle this event
I. Discontinuation of trazodone due to intolerance or adverse event	These discontinuations will mostly occur early, in about 15% of participants, who will then continue in the study via telephone follow-up. Will be addressed in the same way as Event H: **treatment policy**
J. Discontinuation of memantine due to lack of efficacy	The **treatment policy** strategy will be used, as this event is expected to be rare: perceived lack of efficacy withdrawal rare in literature (2% of withdrawals) as is progression (3% of withdrawals)
K. Discontinuation of trazodone due to lack of efficacy	The **treatment policy** strategy will be used, as this event is expected to be rare: perceived lack of efficacy withdrawal rare in literature (2% of withdrawals) as is progression (3% of withdrawals)
L. Participant entering end of life care resulting in truncated data	This will generally happen shortly before end of life, and therefore only one or two ALS-FRS-R measurements will be missing. The ALS-FRS-R scores prior to withdrawal will likely reflect deterioration in condition and a **hypothetical strategy** will be used assuming that end-of-life care or death does not occur
M. Use of memantine in placebo group	The **treatment policy** strategy will be used, as this event is expected to be extremely rare
N. Use of trazodone in placebo group	The **treatment policy** strategy will be used, as this event is expected to be extremely rare
O. Implementation of life extending treatment—gastrostomy	Enteral feeding—affects quality of life rather than survival and occurs in 10–20% of patients. Gastrostomy extends life by about 3 months based on data in Gorrie et al. [23]. As the trial is taking a treatment policy perspective, this event will be handled by using a **treatment policy** strategy
P. Implementation of life extending treatment—ventilation	Trachaeostomy extends life but is very rare. Non-invasive ventilation (NIV) via a mask is more common, occurring in 5% of patients soon after diagnosis and overall 10–20% would be expected over 18 months. NIV does not affect whether ALS-FRS-R can be measured. This event will be dealt with in the same way as Event O: **treatment policy**

#### Estimand for the survival co-primary outcome

For the survival co-primary outcome measure, the estimand population is the same as for the ALS-FRS-R outcome except that long survivors at baseline will be included. The variable is defined as “survival time from randomisation until death”, and the population-level summary is the hazard ratio between each investigational treatment and placebo.

Intercurrent events have been identified which may change the interpretation of the measured survival co-primary outcome, and these are listed in Table 4.

**Table 4:** Intercurrent events for survival co-primary endpoint

Event	Strategy
A. Stopping riluzole during study	Riluzole will extend the life of patients by 2–3 months on average. Audit data suggest that 15–20% of participants will stop riluzole during the course of MND-SMART. As the trial is taking a pragmatic treatment policy approach, this event will be handled using a **treatment policy** strategy
B. Starting riluzole during study	As riluzole treatment is generally initiated close to diagnosis it is expected that few participants (< 10%) will start riluzole during MND-SMART. Again, as the trial is taking a pragmatic treatment policy approach, this event will be handled using a **treatment policy** strategy
C. Concomitant illness unrelated to MND condition	This may reduce survival compared to what it would be if the patient did not have the concomitant illness. A **treatment policy** strategy will be used to handle this event
D. Discontinuation of memantine due to intolerance or adverse event	These discontinuations will mostly occur early, in about 10% of participants, who will then continue in the study via telephone follow-up. The treatment policy perspective of MND-SMART means that a **treatment policy** strategy can be used to handle this event
E. Discontinuation of trazodone due to intolerance or adverse event	These discontinuations will mostly occur early, in about 15% of participants, who will then continue in the study via telephone follow-up. Will be addressed in the same way as Event D: **treatment policy**
F. Discontinuation of memantine due to lack of efficacy	The **treatment policy** strategy will be used, as this event is expected to be rare: perceived lack of efficacy withdrawal rare in literature (2% of withdrawals) as is progression (3% of withdrawals)
G. Discontinuation of trazodone due to lack of efficacy	The **treatment policy** strategy will be used, as this event is expected to be rare: perceived lack of efficacy withdrawal rare in literature (2% of withdrawals) as is progression (3% of withdrawals)
H. Use of memantine in placebo group	The **treatment policy** strategy will be used, as this event is expected to be extremely rare
I. Use of trazodone in placebo group	The **treatment policy** strategy will be used, as this event is expected to be extremely rare
J. Implementation of life extending treatment—gastrostomy	Enteral feeding—affects quality of life rather than survival and occurs in 10–20% of patients. Gastrostomy extends life by about 3 months based on data in Gorrie et al. al. [23] As the trial is taking a treatment policy perspective, this event will be handled by a **treatment policy** strategy
K. Implementation of life extending treatment—ventilation	Trachaeostomy extends life but is very rare. Non-invasive ventilation (NIV) via a mask is more common, occurring in 5% of patients soon after diagnosis and overall 10–20% would be expected over 18 months. NIV does not affect whether ALS-FRS-R can be measured. This event will be dealt with in the same way as Event J: **treatment policy** strategy

### List of statistical analyses

#### Recruitment and retention

The information necessary to construct a Consolidated Standards of Reporting Trials (CONSORT) flow diagram will be provided [24, 25]. This will show the numbers of participants randomised to each group along with the numbers with missing primary outcome data at each measurement time point. For European Union Drug Regulating Authorities Clinical Trials Database (EudraCT) [26] reporting purposes, enrolment will also be summarised into age categories 18–64, 65–84, and 85 + years.

The number and percentage of participants who were later found to be ineligible for the trial even though they were randomised will be summarised by randomised group, as will the number of participants formally withdrawn, the type of withdrawal (e.g. full trial or withdrawal from attending visits etc.), the approximate timing of withdrawal, and the reason for withdrawal (if available). No formal statistical testing will be performed.

Protocol violations are defined as any change, divergence, or departure from the study design, procedures defined in the protocol or Good Clinical Practice (GCP) that may potentially significantly impact the completeness, accuracy, and/or reliability of the study data or that may significantly affect a subject’s rights, safety, or well-being. All such protocol violations will be listed. Protocol deviations are defined as not significantly affecting a subject’s rights, safety, or well-being, or study outcomes. These will be reported only where necessary to enable comprehensive interpretation of the trial data.

The number and percentage of participants for whom the blind was broken early (emergency unblinding events) will also be reported by randomised treatment group.

#### Baseline characteristics

The analyses in this section will be based on analysis population A (full analysis set, including long survivors).

Table 5 shows the baseline characteristics that will be summarised by treatment group and overall.

**Table 5:** Baseline characteristics

Variable	Type of variable
Age (years)	Continuous
Age (by EudraCT reporting categories)	Categorical (18–64, 65–84, 85 +)
Sex	Categorical (M/F)
MND subtype	Categorical (amyotrophic lateral sclerosis, primary lateral sclerosis, progressive muscular atrophy, progressive bulbar palsy, other)
Site of onset	Categorical (bulbar, spinal, respiratory, other)
El Escorial Criteria Level	Categorical (suspected, probable, probable laboratory supported, definite, possible, other, not applicable)
Symptom duration (time since first symptom)	Continuous
Time since diagnosis (time since date of diagnosis)	Continuous
Long survivor (> 8 years since diagnosis)	Categorical (Y/N)
Genetic (%)	Categorical (% of pts with known genetic cause)
Riluzole use	Categorical (Y/N)
NIV and/or gastrostomy	Categorical (Y/N)
ALS-FRS-R	Continuous (total score)
FVC	Continuous
King’s stage	Categorical (1, 2, 3, 4)
EQ-5D-5L	Continuous (index and VAS)
HADS	Continuous (including anxiety and depression subscales, and overall score)
ECAS	Continuous (total participant score, participant score stratified by domains, total carer score)

Categorical variables will be summarised using frequencies and percentages; continuous variables will be summarised using the mean, standard deviation (SD), median, lower quartile, upper quartile, and minimum and maximum values.

This will be done for each interim analysis separately and for the final analysis.

Additionally, for the final analysis only, baseline data summaries will be reported stratified according to whether participants have missing and non-missing follow-up data; and separately, stratified according to long survivor status at baseline.

#### Interim analysis 1

Interim analysis 1 will be based on analysis population B (full analysis set, no long survivors).

A normal linear mixed model will be fitted to the ALS-FRS-R outcome as described in the “Statistical model for the analysis of ALS-FRS-R data” section. The corresponding 95% confidence interval for the difference in slopes for each treatment versus placebo comparison (interaction term (ii) in the model outlined in the “Statistical model for the analysis of ALS-FRS-R data” section) will be calculated. Our interest is in whether a 25% improvement in the rate of decline observed in the placebo arm is below the upper limit of a 95% confidence interval. Higher values of ALS-FRS-R correspond to better functional outcomes, and so a positive value of the difference in slopes implies a reduced decline in ALS-FRS-R over time and a better patient outcome.

To calculate the rate of decline in the placebo arm, we will extract the time coefficients from the model and their corresponding standard errors, before using these data to then calculate an average slope for the time effect in the placebo arm by fitting a linear regression model to the time coefficients, with the time factor variable as a linear explanatory variable, weighted by the inverse standard errors of the time coefficients.

The coefficient for time in this model will then be multiplied by 0.25, and the value compared to the upper limit of the 95% confidence interval for each interaction term. If the upper limit is below the value of 25% improvement for each treatment versus placebo comparison, the treatment will not continue to stage 2. Otherwise, if the upper limit is above the value for 25% improvement, the treatment *will* proceed to stage 2.

#### Interim analysis 2

Interim analysis 2 will be based on analysis population B (full analysis set, no long survivors).

The same normal linear mixed model will be fitted as described in the “Statistical model for the analysis of ALS-FRS-R data” section, except continuation of each investigational treatment to the next stage will be based on the statistical significance of the improvement in the rate of change in ALS-FRS-R compared to placebo, based on interaction term (ii) in the model, using a one-sided 10% significance level.

#### Stage 3 ALS-FRS-R analysis

The stage 3 analysis of ALS-FRS-R will be based on analysis population B (full analysis set, no long survivors). The same normal linear mixed model will be fitted as described in the “Statistical model for the analysis of ALS-FRS-R data” section, with the addition of a factor variable for trial stage included as a covariate in the model to indicate in which stage the participant was randomised into. Specifically, participants randomised before a final decision was made based on the results of the first stage analysis will be regarded as being in stage 1. Participants randomised after the first stage analysis but before a final decision was made based on second stage analysis will be regarded as being in stage 2. Participants randomised after a final decision was made based on the second stage analysis will be regarded as being in stage 3. If there are any changes to the study design made after the results of each analysis are reported, then participants will be divided according to when changes were *implemented* rather than according to when each decision was made.

For each treatment versus placebo comparison, we will report the overall treatment effect (mean difference in rate of change), 95% confidence interval, and *p*-value.

#### Dealing with intercurrent events and missing data for ALS-FRS

For the ALS-FRS-R interim analyses and final analysis, all intercurrent events will be analysed as per the estimand described in the “Estimands” section.

No missing data imputation will be performed for the primary analysis. In sensitivity analysis, we will impute missing values *prior* to death using a multiple imputation (MI) method.

In particular, we will impute missing data using the method of Fully Conditional Specification (FCS) [27] (including treatment arm and all covariates as in the final analysis model), fit the primary analysis model to each imputed dataset, and use Rubin’s rules [28] to combine results for inference. Twenty imputed datasets will be constructed. Note that in all cases, the ALS-FRS score cannot be below 0 or above 48, and so the imputed values will always be restricted to the range 0 to 48. If any values below 0 or above 48 are imputed, then these will be changed to 0 or 48 respectively.

For the primary analysis, we will assume all missing data is “missing-at-random”. However, in further sensitivity analysis, we will use a δ-based MI method [29] under the following conditions:

- If the participant has died during follow-up and there is a continuous sequence of missing data prior to death (e.g. because the participant has been moved to end-of-life care), then we will subtract *δ* = 10% of the observed mean value of ALS-FRS-R in the placebo arm at the relevant time points from the corresponding values imputed under MI before death. No explicit imputations will be made after death.
- If the participant has withdrawn from the trial and withdrawals are known to be due to poor health, concomitant illness, end-of-life care, or deterioration in condition, then we will subtract *δ* = 10% of the mean value of ALS-FRS-R in the placebo arm at the relevant time points from the values imputed under MI.
- If the missing data is continuous after a certain time point (e.g. if the participant records no valid data after month 6 because they have withdrawn from the trial) and the reason for missing data is unknown, then we will subtract *δ* = 5% of the mean value of ALS-FRS in the placebo arm from the values imputed under MI.
- Any other types of missing data not covered above occurring before death and before the participant has reached the relevant clinic visit dates will be imputed based on a missing-at-random assumption.

We will explore how high the *δ* values used in the *δ*-based MI method need to be (a “tipping point analysis”) in order to change the clinical interpretation of the co-primary outcome analysis result, based on a sensitivity analysis method suggested in White et al. [30], Cro et al. [29], and Yan et al. [31]. This secondary analysis will enable us to assess the sensitivity of the results to our assumptions about the strength of deterioration. Imputed values will always be restricted to the range 0 to 48.

#### Primary analysis of the survival co-primary outcome

In the event of a statistically significant result of the ALS-FRS-R co-primary outcome, formal inferential analysis of the survival co-primary outcome will be performed. This analysis will be based on analysis population A (full analysis set, including long survivors).

This analysis will only be done after the final ALS-FRS-R analysis at the end of stage 3 is complete and after at least 113 deaths have been observed in the placebo arm (end of stage 4).

Survival will be calculated as the time from randomisation until death (or until date last known to be alive).

Kaplan–Meier statistics will be used to estimate the survival function and the median survival will be calculated for each randomised group with 95% confidence intervals.

A log-rank test will be used to compare treatment and placebo survival curves for each comparison.

A Cox proportional hazard model including treatment as a factor variable and adjusting for all three minimisation variables (Riluzole, NIV and/or gastrostomy, and long survival) will formally estimate treatment effects. The proportional hazards assumption will be checked using graphical methods: plotting the Kaplan Meier survival curve stratified by treatment arm. Treatment effects will be reported as hazard ratios with 95% confidence intervals.

For the survival analysis outcome, all missing death outcome data will be censored at the date last known alive (e.g. date of withdrawal). We think that missing death information would be very unlikely to occur in practice and that simple censoring at the time of withdrawal would be appropriate. Besides, the Cox proportional hazards regression modelling approach is still valid in the case of competing risks such as participant withdrawal since it allows us to estimate the instantaneous risk of death at time *t* given that the participant has not withdrawn at time *t* [32–34].

#### Secondary outcome analyses

Secondary outcomes will be inferentially analysed after stage 3 only, unless a treatment has stopped early on the basis of an interim analysis, in which case secondary outcomes will be analysed at that point.

Secondary outcomes will be analysed based on analysis population A (full analysis set, including long survivors) and separately using analysis population B (full analysis set, no long survivors).

Missing data will be dealt with in the same way as in the primary analysis.

Analysis will be done separately for each individual time point (e.g. 6, 12, and 18 months) where the outcome has been measured.

A linear mixed effects regression model will be fitted to each continuous secondary outcome at each time point. The following explanatory variables will be included:

(i) Treatment as a factor variable (placebo will be the reference category and two dummy variables for the active treatments)
(ii) Riluzole (baseline minimisation variable)
(iii) NIV and/or gastrostomy (baseline minimisation variable)
(iv) Long survivor status (baseline minimisation variable)
(v) Random intercept for participant

The same Cox proportional hazards survival analysis as in the “Primary analysis of the survival co-primary outcome” section will be performed on time-to-event secondary outcomes (time from randomisation to King’s stage 4A and time to King’s stage 4B) as was done for the survival co-primary outcome. As in the survival analysis, missing data will be censored.

No multiplicity adjustment will be applied to any of these secondary analyses. Secondary outcome results will be interpreted precisely [35] to avoid the impact of an inflated FWER across all secondary outcomes. This means that they will be interpreted in a way that refers to all of their distinguishing features (e.g. time point, type of outcome, intervention) as appropriate, so that we can differentiate clearly between underlying individual hypothesis tests and confidence intervals [35]. These will be considered subsidiary to the primary outcome results.

#### Adverse events

The analyses in this section will be based on analysis population C (safety population, including long survivors) and reported according to treatment received.

All adverse events, serious adverse events, and suspected unexpected serious adverse reactions (SUSARs) will be listed, stratified by treatment arm.

The number and percentage of adverse events will be reported, per treatment arm and overall, as well as the number and percentage of *participants with at least one adverse event*.

Similarly, the number and percentage of serious adverse events (and number and percentage of participants with at least one serious adverse event) will be reported by treatment arm and overall.

In addition, we will report the above tabulations of numbers of adverse events (and participants with at least one adverse event) subdivided by system organ class MedDRA classification [36].

#### Subgroup analysis

We will also perform pre-specified sub-group analyses on particular participant and disease characteristics to explore any effect of the heterogeneous study population on therapeutic benefit of the investigational treatments.

These subgroup analyses will only be done at the end of stage 4 unless a treatment stops early at an interim analysis, in which case this analysis will take place at the point of stopping.

Pre-planned subgroup analyses of co-primary and secondary outcome measures will be performed for the list of variables below. These analyses will involve adding treatment-by-subgroup interaction terms to the same models as used to analyse the primary and secondary outcomes as detailed above and assessing the statistical significance of these interaction terms. No sub-domains of secondary outcomes will be analysed: we will only analyse total or overall scores of secondary outcomes within the subgroup analyses. For the co-primary outcome ALS-FRS-R model, subgroup analysis will involve including a main effects subgroup factor term, subgroup-by-time interaction terms, and a subgroup-by-treatment-by-time interaction term. For the survival co-primary outcome model and secondary outcome models, this will involve including a main effects subgroup factor term, and a subgroup-by-treatment interaction term. Analysis populations will remain the same. As before, these analyses will be based on analysis population B (full analysis set, no long survivors) for the ALS-FRS-R outcome and analysis population A (full analysis set, with long survivors) for the survival outcome and secondary outcomes.

The list of variables used for the subgroup analyses is as follows:

1. Survival at baseline (≥ 8 years, < 8 years)
2. Sub-types of MND (including amyotrophic lateral sclerosis, primary lateral sclerosis, and progressive muscular atrophy)
3. Participants with and without C9orf72 expansions
4. Participants with and without bulbar onset and limb onset forms of MND
5. Age (< 40 years, 40–80 years, 80 + years)
6. Participants receiving non-invasive ventilation support at randomisation
7. Participants receiving support with gastrostomy feeding at randomisation

#### Descriptive statistics of outcome variables

Descriptive statistics will be reported for the ALS-FRS-R outcome, all continuous secondary outcomes, and the individual items of EQ-5D-5L, stratified by treatment arm and clinic visit. Note that the descriptive analysis of ALS-FRS-R outcome will include measurement time points after 18 months. A line plot will be drawn to show the change in mean ALS-FRS-R score over time, stratified by treatment arm.

These analyses will be based on analysis population A (full analysis set, with long survivors). For the ALS-FRS-R outcome, separate sets of descriptive statistics will be calculated based on analysis population A *and* analysis population B (full analysis set, no long survivors).

#### Medication adherence

Participants will be asked to record adherence to allocated treatment using a diary card to record the dose of drug taken and to indicate any reason for non-adherence.

The numbers and percentage of participants considered to be “adherent to medication” will be reported in each randomised treatment arm at the final analysis stage. Participants will be considered to have followed treatment as planned unless they report failing to take their study drug for three consecutive days or 10 days in total in the 60 days preceding each visit.

This analysis will be based on analysis population A (full analysis set, including long survivors).

#### Concomitant medications and riluzole use

The numbers and percentage of participants using each type of concomitant medication at least once during the trial will be reported split by randomised treatment group. Infrequent or rare concomitant medications will be grouped into an “Other” category.

Of particular interest will be the number and percentage of participants using riluzole, split by treatment arm. We will also separately report the number of participants who started using riluzole during the trial when they were not using it at baseline.

These analyses will be based on analysis population A (full analysis set, including long survivors).

#### Data sharing

MND-SMART has a trial specific process for data sharing, and data sharing requests must be made by formal application to the trial team. Requests for unblinded data will be reviewed by the Trial Management Group each month and will be escalated to the Trial Steering Committee chair as necessary. Unblinded data will only be shared while the trial is in progress if it does not compromise the validity, blinding, and integrity of the MND-SMART trial. In particular, the sharing of longitudinal data is unlikely to be appropriate while the trial is in progress. After the trial is finished, data requests will be reviewed by the trial team and will not be refused without good reason.

If circumstances change, information emerges, or issues emerge which were not anticipated when this SAP was written we reserve the right to alter this SAP. We shall ensure any such changes will be madeindependently of the accumulating results from the trial and preserve the trial integrity.

## Discussion

Construction of the statistical analysis plan for MND-SMART required detailed consideration of issues relating to multiple testing and estimand specification due to the multi-arm multi-stage nature of the trial, the inclusion of co-primary outcomes measuring functional outcome (ALS-FRS-R), and survival and the repeated measurements of ALS-FRS-R. The analysis plan outlined above attempts to balance multiple factors, including minimisation of bias, maximising power and precision, and deriving clinically interpretable summaries of treatment effects. At the same time, it retains a pragmatic perspective to ensure an analysis that is readily deliverable within the additional constraints of a trial design featuring multiple interim analyses.

Moving from control of the familywise error rate to control of the pairwise error rate was acceptable to the MHRA and helped to improve trial efficiency.

This decision resulted in a substantial reduction in sample size, which is very important given the rapidly progressive and fatal nature of MND. Many participants joining the trial unfortunately will not live to see the results. Our aim was to reach definitive answers as quickly and efficiently as possible.

## Trial status

This trial is currently in active recruitment. The first participant was randomised on 27th February 2020.

## Supplementary Information

**Additional file 1.** Simulation work for co-primary ALS-FRS-R analysis model development.

**Additional file 2.**

## Data Availability

After the MND-SMART trial is finished, data requests will be reviewed by the trial team and will not be refused without good reason.

## Abbreviations

ACORD: A Collaboration Of groups developing, Running and reporting multi-arm multi-stage trials in neurodegenerative Diseases
ALS: Amyotrophic lateral sclerosis
ALS-FRS-R: Amyotrophic Lateral Sclerosis Functional Rating Scale Revised
CI: Confidence interval
CONSORT: Consolidated Standards of Reporting Trials
ECAS: Edinburgh Cognitive and Behavioural ALS Screen
EudraCT: European Union Drug Regulating Authorities Clinical Trials Database
FCS: Fully conditional specification
FVC: Forced vital capacity
FWER: Familywise error rate
GEE: Generalised estimating equations
HADS: Hospital Anxiety and Depression Scale
ICH: International Council for Harmonisation of Technical Requirements for Pharmaceuticals for Human Use
MAMS: Multi-arm multi-stage
MHRA: Medicines and Healthcare products Regulatory Agency
MI: Multiple imputation
MND-SMART: Motor Neuron Disease Systematic Multi-Arm Adaptive Randomised Trial
MND: Motor neuron disease
NIV: Non-invasive ventilation
PRO-ACT: Pooled Resource Open-Access ALS Clinical Trials
SD: Standard deviation
SUSARs: Suspected unexpected serious adverse reactions
UK: United Kingdom
