# Supplementary material for: Statistical analysis plan for the motor neuron disease systematic multi-arm adaptive randomised trial (MND-SMART)
Source: Trials. 2023 Jan 16;24:29. doi: 10.1186/s13063-022-07007-z (PMC9843918; doi:10.1186/s13063-022-07007-z)
Supplement: Supplementary file 1 — Additional file 1. Simulation work for co-primary ALS-FRS-R analysis model development. [file 13063_2022_7007_MOESM1_ESM.docx]

**Statistical Analysis Plan for the Motor Neuron Disease Systematic Multi-Arm Adaptive Randomised Trial (MND-SMART)**

**Supplementary File 1**

**Simulation work for co-primary ALS-FRS-R analysis model development**

1. **AIM**

To explore different models for the ALS-FRS-R analysis.

1. **DATA GENERATING PROCEDURE**

We considered 3 cases regarding the treatment effect:

- Case (a): treatment affects neither ALS-FRS-R nor survival;
- Case (b): treatment affects ALS-FRS-R but not survival;
- Case (c): treatment affects both ALS-FRS-R and survival via a shared random effect.

*Case (a) Case (b) Case (c)*


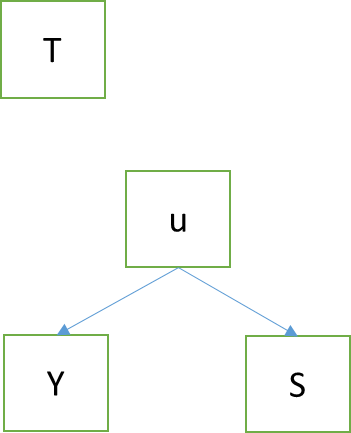

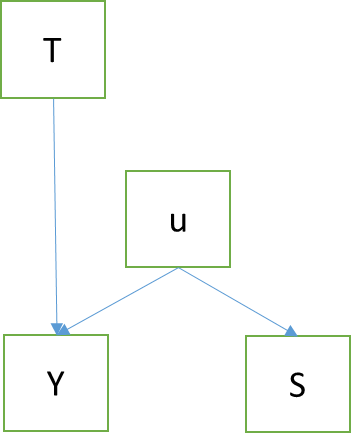

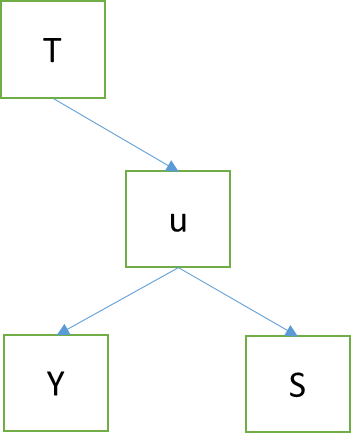


*T, treatment; Y, outcome; S, survival; u, random slope*

For each of the n_sample_ = 200 participants:

- Generate a randomised treatment trt with probability 0.5;
- Generate random coefficients for participant ALS-FRS-R trajectory b_intercept ~ N(30, 1^2^) and b_visit;
- Cases (a) and (b): b_visit ~ N(-0.9, 0.8^2^)
- Case (c): b_visit ~ N(0.7trt - 0.9, 0.8^2^)
- Generate individual deviation from the mean slope b_visit_randerr = b_visit - (-0.9)
- Generate survival time and event indicator with administrative censoring at 5 years using the Stata user-written command survsim^1^ (mortality at 5 years ~50%)
  - Cases (a) and (b): exponential baseline hazard with λ = 0.13; covariate b_visit_randerr with log hazard ratio = -0.3
    - Stata code: survsim stime died, dist(exponential) lambda(0.13) cov(b_visit_randerr -0.3) maxtime(5);
  - Case (c): exponential baseline hazard with λ = 0.2; covariate b_visit_randerr with log hazard ratio = -1.5
    - Stata code: survsim stime died, dist(exponential) lambda(0.13) cov(b_visit_randerr -0.3) maxtime(5);
- Expand the data so each participant has 5 rows of data for 1 baseline and 4 follow-up visits;
- Generate individual-level random errors e ~ N(0, (√3)^2^);
- Generate individual ALS trajectories; ALS scores decline over time
- Cases (a) and (c): als = b_intercept + b_visit*visit + e;
- Case (b): als = b_intercept + b_visit*visit + 0.5*trt*visit + e;
- Generate variables start and stop for each observation window (the number of rows per participant now vary, depending on when they die);
- Update event (death) indicator for visits during which the participants are still alive
- Generate baseline ALS-FRS-R score als_bs for each participant.
- Repeat steps 1–10 to create n_rep_ = 1,000 simulation repetitions.

1. **ESTIMAND**

Treatment effect on ALS-FRS-R score at the last visit.

1. **METHODS OF ANALYSIS**

Stata code

Unconditional estimand

Mixed model 1: time-specific treatment effect

mixed als i.visit 1.trt#i.visit i.visit#c.als_bl if visit > 0 || id: visit, stddev cov(uns) iter(20) reml dfmethod(kroger)

Mixed model 2: treatment effect is linear with time

mixed als i.trt i.visit 1.trt#c.visit c.als_bl i.visit#c.als_bl if visit > 0 || id: visit, stddev cov(uns) iter(20) reml dfmethod(kroger)

Mixed model 3: treatment effect is proportional to time

mixed als i.visit 1.trt#c.visit c.als_bl i.visit#c.als_bl if visit > 0 || id: visit, stddev cov(uns) iter(20) reml dfmethod(kroger)

Partly conditional estimand

GEE model 1: time-specific treatment effect

reg als i.visit 1.trt#i.visit i.visit#c.als_bl if visit > 0, vce(cluster id)

GEE model 2: treatment effect is linear with time

reg als i.trt i.visit 1.trt#c.visit c.als_bl i.visit#c.als_bl if visit > 0, vce(cluster id)

GEE model 3: treatment effect is proportional to time

reg als i.visit 1.trt#c.visit c.als_bl i.visit#c.als_bl if visit > 0, vce(cluster id)

1. **PERFORMANCE MEASURES**

- Bias in point estimate;
- Efficiency (average model standard errors versus empirical standard errors);
- Coverage of 95% confidence intervals;
- Power of 5% test.

1. **RESULTS (graphs)**

Case (a): treatment affects neither ALS-FRS-R nor survival

Unconditional estimand = partly conditional estimand = 0

Case (b): treatment affects ALS-FRS-R but not survival

Unconditional estimand = partly conditional estimand = 2

Case (c): treatment affects both ALS-FRS-R and survival via a shared random effect

Unconditional estimand = 2.016

Partly conditional estimand = 1.861

**REFERENCES**

1. Crowther, M. J. & Lambert, P. C. Simulating complex survival data. *Stata J.* **12**, 674–687 (2012).
